# UL45–46 as the preferred insertion site for herpesvirus-of-turkeys-vectored H9N2 avian influenza vaccines: comparative analysis of immunogenicity and protection

**DOI:** 10.1186/s13567-026-01850-7

**Published:** 2026-11-13

**Authors:** Wenhao Yang, Ruili Su, Jin Zhang, Fengmin Zhang, Sige Wang, Xiaolong Lu, Ruyi Gao, Kaituo Liu, Yu Chen, Min Gu, Shunlin Hu, Xiaowen Liu, Xiaoquan Wang, Xiufan Liu

**Affiliations:** 1 Key Laboratory of Avian Bioproducts Development, Ministry of Agriculture and Rural Affairs, College of Veterinary Medicine, Yangzhou University, 48 East Wenhui Road, Yangzhou, 225009 Jiangsu China; 2 Jiangsu Co-innovation Center for Prevention and Control of Important Animal Infectious Diseases and Zoonoses, Yangzhou University, Yangzhou, 225009 China; 3 Jiangsu Interdisciplinary Center for Zoonoses and Biosafety, Yangzhou University, Yangzhou, 225009 China; 4 Jiangsu Key Laboratory of Zoonosis, Yangzhou University, Yangzhou, 225009 China; 5 Joint International Research Laboratory of Agriculture and Agri-Product Safety, The Ministry of Education of China, Yangzhou University, Yangzhou, 225009 China

**Keywords:** Herpesvirus-of-turkeys, H9N2, UL45–46, US2

## Abstract

H9N2 avian influenza virus (AIV) has evolved into a threat to the global poultry industry, causing huge economic losses and persistent public health risks. Due to inadequate cellular and mucosal immunity, current inactivated vaccines fail to prevent infection and block virus transmission. The recombinant herpesvirus-of-turkeys (HVT)-vectored vaccine is a promising control strategy, but the insertion site with the higher immunogenicity of the hemagglutinin (HA) gene is still unclear. This study compared the immunogenicity and protective efficacy of recombinant HVT vaccines expressing the H9N2 HA gene at the unique long (UL)45–46 and unique short (US)2 sites. The HVT-HA^UL45–46^ virus maintained replication kinetics consistent with the parental HVT and demonstrated genetic stability through 20 consecutive passages. Compared with HVT-HA^US2^, HVT-HA^UL45–46^ induced significantly higher hemagglutination inhibition (HI) antibody titers and stronger HA-specific cellular immunity. Following the H9N2 challenge, the UL45–46 vaccine completely prevented intestinal viral shedding and significantly reduced lung viral loads and histopathological lesions. These findings establish UL45–46 as a better insertion site for HVT-based H9N2 vaccines.

**Supplementary information:**

The online version contains supplementary materials available at https://doi.org/10.1186/s13567-026-01850-7.

## Introduction

Since the 1990s, the H9N2 subtype avian influenza virus (AIV) has evolved from a sporadically occurring low-pathogenic pathogen into an entrenched endemic disease in the global poultry industry. Especially in Asia, the Middle East, North Africa, and recently in parts of Europe, the ongoing spread of H9N2 viruses has caused huge economic losses and posed a continuous threat to public health [1]. However, the H9N2 virus usually shows low-pathogenicity avian influenza (LPAI) and relatively mild clinical symptoms. In intensive farming environments, H9N2 infection in chickens often leads to a significant decrease in egg production, slower growth in broilers, and reduced feed conversion rates. More importantly, H9N2 infection induces severe immunosuppression, damages the respiratory mucosal barrier, and makes poultry highly susceptible to secondary coinfections with other pathogens such as *Escherichia coli* and infectious bronchitis virus (IBV), resulting in a sharp increase in mortality rates [2].

In South Asia, particularly Bangladesh, H9N2 has been circulating in poultry flocks since 2006, causing ongoing outbreaks and huge economic losses [1, 3]. Studies have shown that in Bangladesh’s live poultry markets (LPMs), H9N2 has become the main LPAI subtype and frequently cocirculates with the highly pathogenic H5N1 virus, facilitating complex reassortment events within poultry trading networks [4]. Meanwhile, the epidemiological situation in West Africa is developing rapidly. Since it was first detected in Burkina Faso in 2017, H9N2 has spread across the region, including to Mali and Ghana [5, 6]. However, due to limited basic surveillance infrastructure, the precise transmission dynamics and genetic evolution of H9N2 in West Africa have not been fully explored, presenting a significant gap in global epidemiological monitoring [7]. In East China, monitoring data indicate that the host range of H9N2 is continuously expanding. H9N2 has been detected not only in chickens, but also from green peafowls and various wild bird species, suggesting the risk of interspecies transmission [8].

H9N2 poses a public health threat far beyond the poultry industry because it plays a key role as a “gene donor” in the AIV reassortment. H9N2 frequently provides internal gene cassettes to other subtypes, facilitating the emergence of new, potentially pandemic reassortant strains. This mechanism has been repeatedly identified, including in H7N9, H10N8, and the recently emerged H3N8 [9, 10]. In addition, surveillance data from 2024 to early 2025 indicate the risk of interspecies transmission of H9N2, particularly to humans, is increasing. According to recent reports from the World Health Organization (WHO) and the European Food Safety Authority (EFSA), sporadic human infections with H9N2 were confirmed across multiple regions, particularly in China and Bangladesh, with a large proportion of cases in children [11, 12]. Molecular studies have shown that these cross-species spillovers are driven by adaptive mutations in the hemagglutinin (HA) protein [13]. The accumulation of such mammalian adaptive mutations positions H9N2 as a candidate pathogen with significant pandemic potential.

Currently, the control of H9N2 avian influenza mainly relies on whole-virus inactivated oil-emulsion vaccines. While these vaccines are effective in reducing mortality and clinical symptoms, they show significant limitations in preventing infection and blocking viral transmission. H9N2 is a mucosal pathogen that primarily targets the respiratory and digestive tracts. Inactivated vaccines administered subcutaneously do not induce effective secretory immunoglobulin A (sIgA) on mucosal surfaces. Thus, even flocks vaccinated with inactivated vaccines continue to shed the virus to the environment [14]. Therefore, the development of novel vaccine platforms capable of inducing cellular and mucosal responses is a priority. The herpesvirus-of-turkeys (HVT) vector has emerged as a promising candidate to address these challenges [15, 16].

There are multiple nonessential genes within the HVT genome [17], most of which could potentially be used as insertion sites, and the gene of interest would have a fundamental effect on virus stability and immune response. Therefore, the “ideal insertion site” may differ depending on the target disease and/or design, such as bivalent or multivalent vaccine design. The structural characteristics of the HVT genome consist of unique long (UL) and unique short (US) regions, providing several nonessential sites, including the intergenic region US2, US10, and UL45–46, which can accommodate the insertion of an exogenous gene [18]. For instance, the UL45–46 intergenic region has been successfully used in commercial HVT/Newcastle disease (ND) vaccines, demonstrating that this site has good antigen stability and immunological protection efficacy [19]. Additionally, compared with the US10 site, inserting the HA gene into the US2 site results in higher antibody titers and protection effects [20]. However, there is currently a lack of assessment of the immunogenicity and protective potential between the commercially validated UL45–46 site and the efficient US2 site.

This study, building upon our laboratory’s previously established HVT-HA^US2^ vaccine strain [16], constructs a recombinant HVT vaccine (HVT-HA^UL45–46^) with the H9N2 avian influenza HA gene inserted at the UL45–46 site. First, the biological properties of the recombinant viruses are systematically characterized through viral titer determination, genomic stability analysis, and in vitro replication efficiency assessment. Subsequently, immunization experiments are conducted in specific-pathogen-free (SPF) chickens to quantify hemagglutination inhibition (HI) titers and HA-specific antibody levels. HA-specific T-cell immune responses are further evaluated using enzyme-linked immunosorbent spot (ELISpot) technology. The study systematically explored the immunogenicity and protective efficacy of HVT-HA differences between the US2 and UL45–46 site.

## Materials and methods

### Viruses and cells

Primary chicken embryo fibroblasts (CEFs) were cultured and maintained in Dulbecco’s Modified Eagle’s Medium supplemented with 10% fetal bovine serum (FBS) at 37 °C in a 5% CO_2_ atmosphere, derived from 9-day-old specific-pathogen-free (SPF) chicken embryos. The HVT-FC126 viral strain was kindly supplied by Dr Witter (United States Department of Agriculture, Avian Disease and Oncology Laboratory). The H9N2 influenza A virus strain A/chicken/Anhui/AH463/2017 (AH463) and HVT-HA^US2^ were obtained from the Animal Infectious Disease Laboratory (School of Veterinary Medicine, Yangzhou University).

### Rescue of HVT-HA^UL45–46^ recombinant virus

The HVT-HA^US2^ strain expressing the HA gene at the US2 site was previously developed and characterized by our group [16]. In the present study, the same technical framework was employed to generate new constructs of HVT-HA^UL45–46^, targeting the UL45–46 intergenic region [21]. Because the purpose of this study was to compare the difference of the immunoprotective effect between US2 and UL45–46 sites, the fluorescent reporter gene of recombinant virus HVT-HA was not excised. Briefly, the genome of HVT was cut at UL45–46 by Clustered Regularly Interspaced Short Palindromic Repeats (CRISPR)/CRISPR-associated protein 9 (Cas9) technology, and then the expression cassette was inserted into this site by nonhomologous end joining (NHEJ). The site-specific integration of the HA gene into the UL45–46 site of the HVT genome was verified by polymerase chain reaction (PCR) using flanking primers (Additional file 1). The stable expression of the HA protein was confirmed through western blot using polyclonal mouse anti-HA antibody (prepared by our lab), and goat anti-mouse IgG secondary antibody, horseradish peroxidase (HRP) (Thermo Fisher Scientific; Waltham, MA, USA). The expression of HA protein was assessed through immunofluorescence assay (IFA) using polyclonal chicken anti-H9N2 serum (prepared by our lab), and goat anti-chicken IgY (H + L) secondary antibody, Alexa Fluor 546 (Thermo Fisher Scientific; Waltham, MA, USA). Following the procedures previously described [16], the in vitro growth kinetics and genetic stability of the recombinant virus were evaluated.

### Experimental design

**Figure 1:**
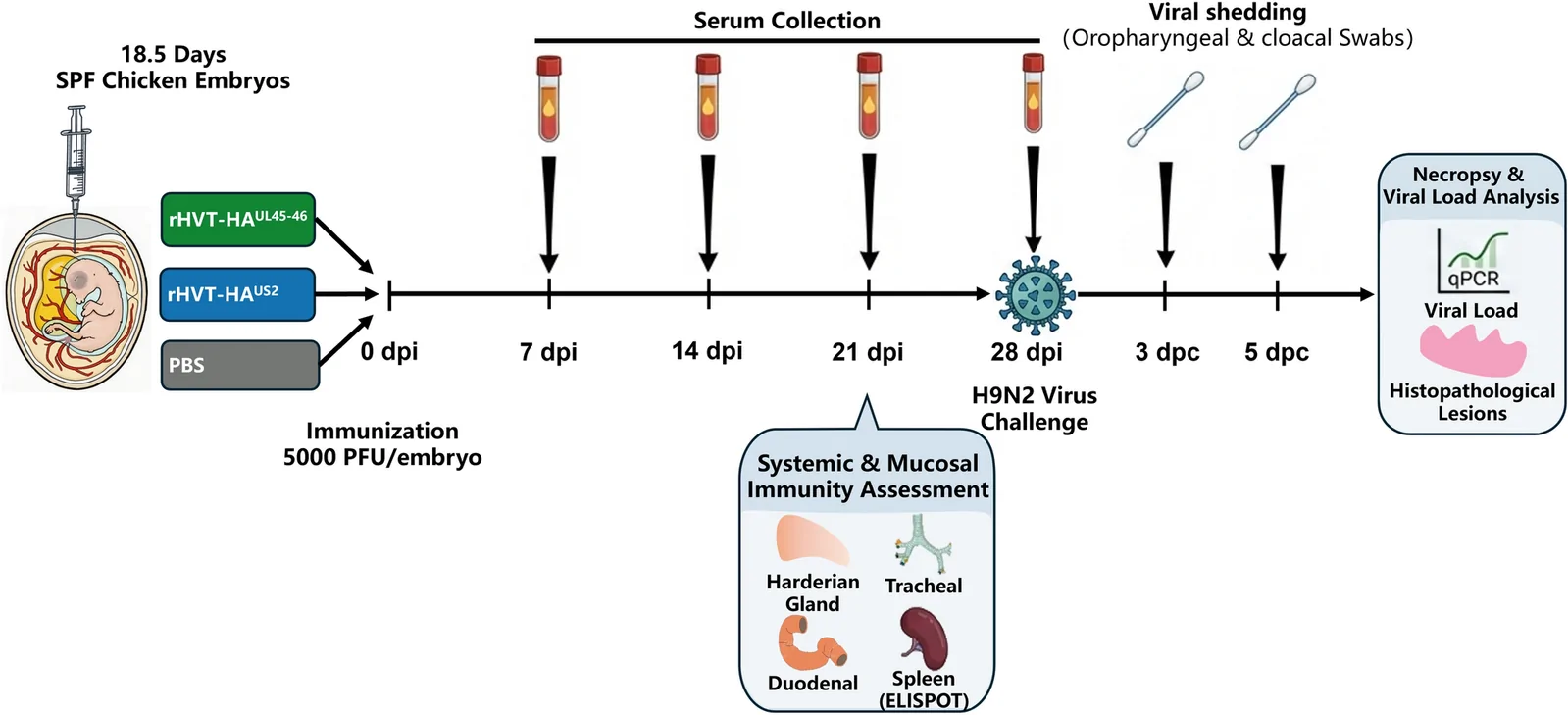
**Schematic representation of the experimental design.**

The workflow of the immunization and challenge experiment is illustrated in Figure 1. A total of 45 SPF chicken embryos were randomly assigned to three groups and inoculated with 5000 plaque-forming units (PFU)/embryo of HVT-HA^US2^, HVT-HA^UL45–46^, or phosphate-buffered saline (PBS) (negative control) using 1 mL syringes. In-ovo vaccination was performed via amniotic cavity injection at 18.5 days of incubation (E18.5). Serum samples were collected from seven randomly selected chickens per group at 7, 14, 21, and 28 days post-immunization (dpi). At 21 dpi, five chickens from each group were euthanized for the collection of harderian glands, tracheal washes, and duodenal washes. Harderian gland tissues were homogenized and centrifuged at 2000–3000 rpm for 20 min at 4 °C, and the supernatants were stored at −20 °C. Tracheal and duodenal washes were similarly processed and stored for antibody detection. Additionally, spleens were harvested for ELISpot assays. At 28 dpi, all chickens were challenged with H9N2 influenza virus (10^4^ EID_50_/0.1 mL, where “EID” is “emerging infectious disease”) via the intranasal and intraocular routes. Oropharyngeal and cloacal swabs were collected at 3 and 5 days post-challenge (dpc) to monitor viral shedding using 9-day-old SPF chicken embryos. At 5 dpc, five chickens from each group were randomly selected and euthanized to harvest lungs. These tissues were used for the quantification of viral loads, and the lung tissues were further processed for hematoxylin and eosin (H&E) staining to evaluate histopathological lesions.

SPF chicken embryos were randomly assigned into three groups: HVT-HA^US2^, HVT-HA^UL45–46^, and PBS control. In-ovo vaccination (5000 PFU/embryo) was performed via amniotic cavity injection at 18.5 days. Sera were collected at 7, 14, 21, and 28 days post-immunization (dpi). At 21 dpi, harderian glands, tracheal, and duodenal washes were harvested for antibody detection. Spleens were harvested for ELISpot assays. At 28 dpi, chickens were challenged with 10^4^ EID_50_ of H9N2 virus. Oropharyngeal and cloacal swabs were collected at 3 and 5 days post-challenge (dpc) to monitor viral shedding. At 5 dpc, lungs were harvested for viral load quantification and histopathological (H&E) analysis.

### Hemagglutination inhibition (HI) and virus neutralization (VN) assays

Systemic and mucosal antibody responses were evaluated using hemagglutination inhibition (HI) and VN assays. For serum samples, both HI and neutralizing antibodies (NAbs) titers against the H9N2 virus were determined to assess humoral immunity. To evaluate mucosal immunity, the Harderian gland homogenates, tracheal washes, and duodenal washes were subjected to VN assays to quantify local NAbs levels. The HI assay was performed following standard protocols using 4 hemagglutinating units (HAU) of the antigen. The NAbs titers for all samples were calculated using the Reed–Muench method.

### ELISpot

Cell-mediated immunity was evaluated using ELISpot. Briefly, polyvinylidene-fluoride (PVDF)-bottomed plates were activated with 35% ethanol and coated with unconjugated anti-chicken interferon (IFN)-gamma monoclonal antibodies (mAb) (MT6C2) (Mabtech, 3125-3-250; 1:100) overnight at 4 °C. After blocking with Roswell Park Memorial Institute (RPMI) 1640 Medium containing 10% FBS for 2 h at 37 °C, 10^6^ splenocytes were added to each well. The cells were stimulated with 100 μL of inactivated H9N2 virus and incubated at 37 °C for 48 h. Splenocytes stimulated with phorbol 12-myristate 13-acetate (PMA; 50 ng/mL) and ionomycin (500 ng/mL) served as the positive control, while cells incubated with RPMI 1640 Medium alone (MOCK) served as the negative control. Subsequently, the plates were washed and incubated with biotinylated anti-chicken IFN-gamma mAb, biotin (Mabtech, 3125-6-250; 1:1000) for 2 h, followed by Streptavidin–alkaline phosphatase conjugate (SA–ALP) (Mabtech, 3310-10-1000; 1:1000) for 1 h at 37 °C. Spots were developed using a chromogenic substrate in the dark and quantified using an ELISpot reader.

### Statistical analysis

All statistical analyses were performed using GraphPad Prism (version 9.0, GraphPad Software Inc.; San Diego, CA, USA). For comparison of multiple groups, one-way analysis of variance (ANOVA) was employed. A *p*-value of < 0.05 was considered statistically significant (^*^p < 0.05, ^**^p < 0.01, ^***^p < 0.001).

## Results

### Generation and characterization of the recombinant HVT-HA^UL45–46^ virus

**Figure 2:**
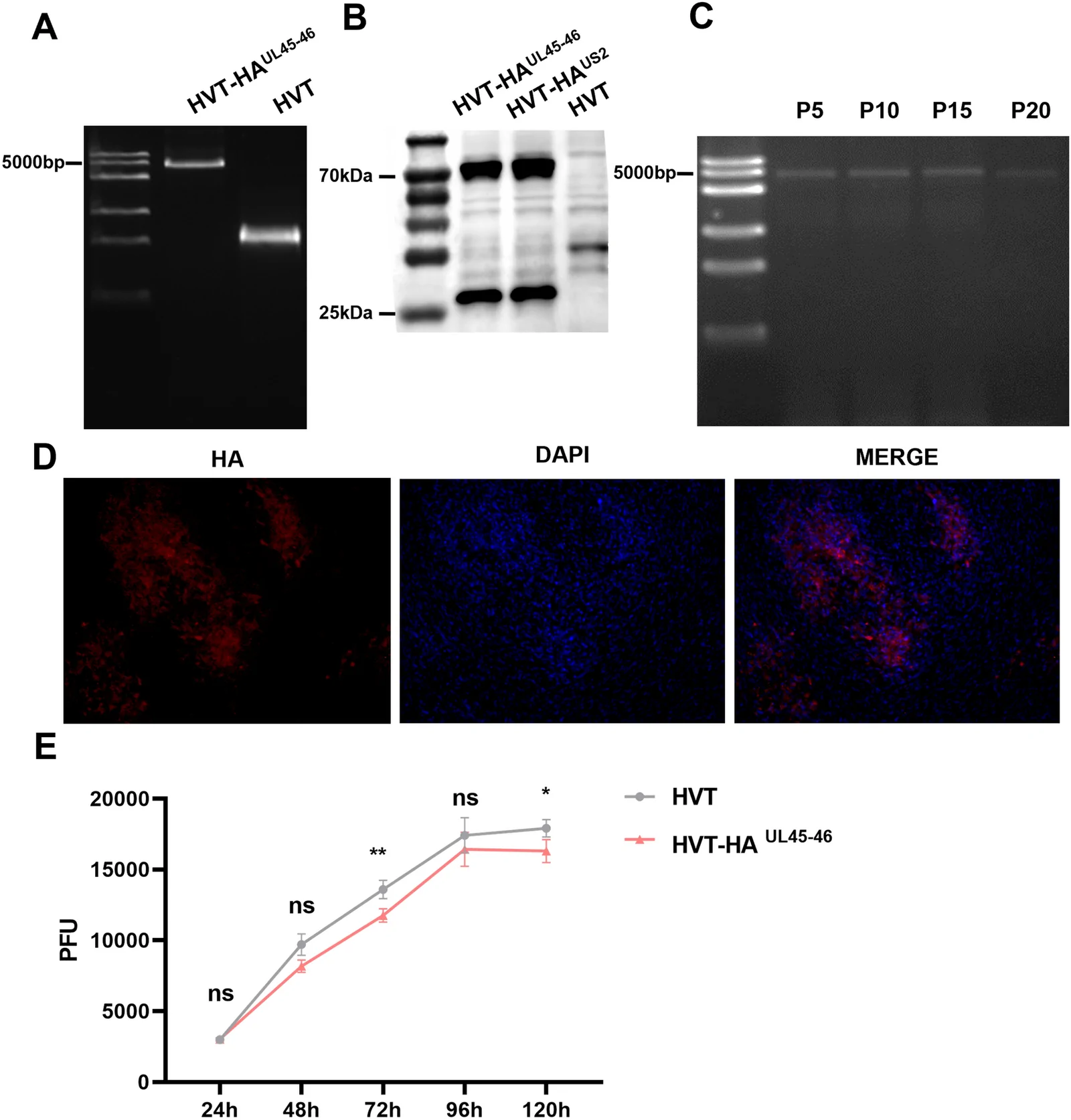
**Characterization of the recombinant HVT-HA**^**UL45–46**^ **virus.** **A** PCR analysis of the HA expression cassette integration at the UL45–46 site of the HVT genome using specific flanking primers. **B** Western blot analysis of HA protein expression in CEF cells infected with HVT-HA^UL45–46^. **C** Genetic stability analysis of the HA gene via PCR amplification across 20 serial passages (P) (P5, P10, P15, and P20) in CEF cells. **D** Immunofluorescence assay (IFA) of HA protein expression (red) in HVT-HA^UL45–46^-infected CEF cells. Nuclei were counterstained with 4′,6-diamidino-2-phenylindole (DAPI) (blue). **E** Multistep growth kinetics of HVT-HA^UL45–46^ and parental HVT in CEF cells. Viral titers were determined at 24, 48, 72, 96, and 120 hours post-infection (hpi). Data are expressed as mean ± standard deviation (SD) (*n* = 3).

The recombinant virus HVT-HA^UL45–46^ was successfully generated by inserting the HA expression cassette into the UL45–46 site of the HVT genome. The PCR assay showed that parental HVT produced shorter bands, whereas the modified virus produced longer bands, confirming the site-specific integration of the HA gene (Figure 2A). Western blot analysis further demonstrated that the HA protein was expressed in HVT-HA^UL45–46^-infected cells with a molecular weight of approximately 70 kDa, consistent with its predicted size (Figure 2B). The indirect immunofluorescence analysis (IFA) results showed fluorescence in the cytoplasm of infected CEF cells, confirming the successful expression of the HA protein (Figure 2D).

### In vitro growth kinetics and genetic stability of HVT-HA^UL45–46^

The genetic stability of HVT-HA^UL45–46^ was evaluated by serially passaging the virus in CEF cells for 20 generations. PCR amplification of the UL45–46–HA region confirmed that the HA gene was stably maintained without any deletions or mutations throughout the passages (Figure 2C, Additional file 2). To determine whether the insertion of the HA gene affected viral replication, multistep growth curves were performed in CEF cells. The replication kinetics of HVT-HA^UL45–46^ were compared with those of the parental HVT. As shown in Figure 2E, the growth performance of parental HVT was slightly higher than that of recombinant virus HVT-HA^UL45–46^, and there was significant difference only at 72 and 120 hours post-infection (hpi). These results indicate that the integration of the HA cassette into the UL45–46 site did not impair the in vitro replication efficiency of the HVT vector.

### Systemic and mucosal antibody responses induced by HVT-HA vaccines

**Figure 3:**
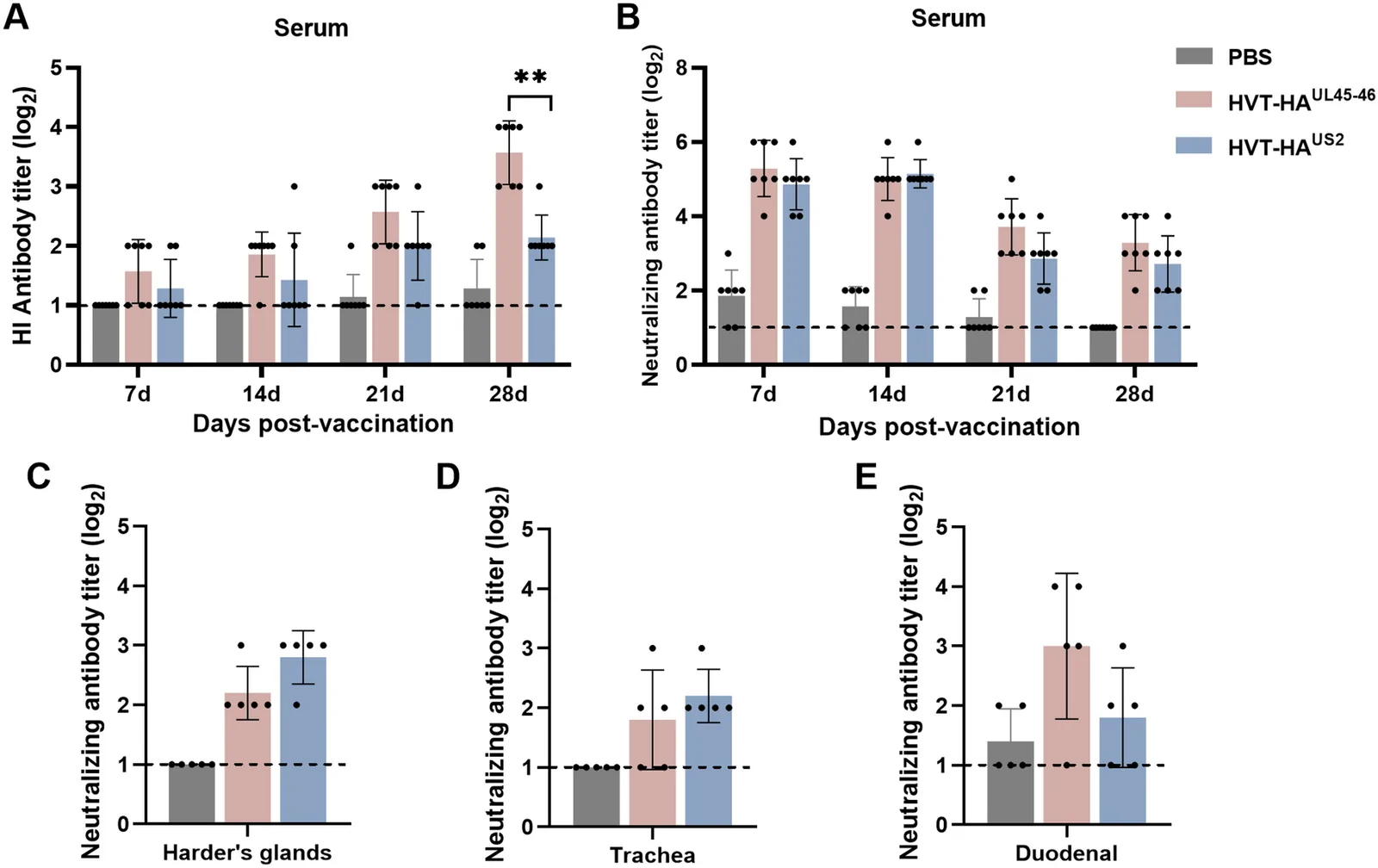
**Systemic and mucosal antibody responses in chickens immunized with HVT-HA vaccines.** H9N2-specific hemagglutination inhibition (HI) **A** and neutralizing antibodies (NAbs) **B** titers were determined in serum samples collected at 7, 14, 21, and 28 dpi. **C**–**E** Neutralizing activity was assessed in mucosal washes/tissues collected from the harderian glands **C**, tracheal washes **D**, and duodenal washes **E**. Data are expressed as mean ± SD.

To evaluate the humoral and mucosal immune responses elicited by the recombinant viruses, serum and mucosal samples were analyzed using HI and VN assays. Both HVT-HA^UL45–46^ and HVT-HA^US2^ successfully induced H9N2-specific HI antibodies from 7 dpi. During the entire 28-day observation period, the titer showed a gradually increasing trend. At 28 dpi, the HI titer of the HVT-HA^UL45–46^ group reached 3.58 ± 0.54 log_2_, which was significantly higher than that of the HVT-HA^US2^ group (Figure 3A). High levels of NAbs were detected in the sera of all vaccinated chickens; however, a gradual declining trend was observed over time. The HVT-HA^UL45–46^ group maintained a relatively higher serum NAbs level, 5.29 ± 0.76 log_2_ at 7 dpi, but no significant difference was observed compared with the HVT-HA^US2^ group (*p* > 0.05) (Figure 3B). Furthermore, both vaccines successfully induced NAbs in the harderian glands, tracheal washes, and duodenal washes. While the HVT-HA^UL45–46^ group exhibited slightly higher NAbs in the duodenum, the difference was not statistically significant compared with the HVT-HA^US2^ group (Figure 3C–E).

### The HVT-HA^UL45–46^-vaccinated group exhibited a robust cellular immune response

**Figure 4:**
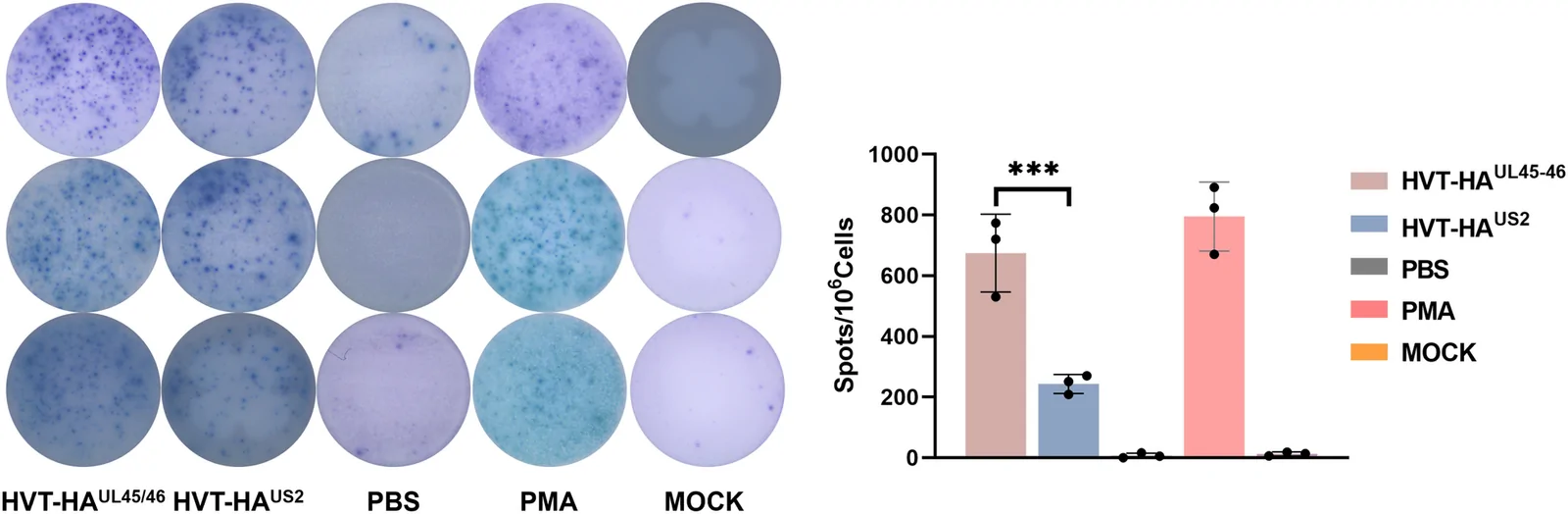
**Antigen-specific cellular immune responses evaluated by the IFN-** ***γ*** **ELISpot assay.** Splenocytes were harvested from immunization groups (HVT-HA^UL45–46^, HVT-HA^US2^, and PBS) and stimulated in vitro with inactivated H9N2 virus. Cells stimulated with PMA or incubated with medium alone (MOCK) were included as the positive control and the assay background control, respectively. The number of spot-forming cells (SFCs) is expressed per 10^6^ splenocytes. Data are presented as mean ± SD (*n* = 3). ^*^ indicates statistical significance between the designated vaccine groups (^***^p < 0.001).

To evaluate the antigen-specific cell-mediated immune responses elicited by the different vaccines, splenocytes were harvested from immunized chickens and stimulated ex vivo with inactivated H9N2 virus to quantify IFN-*γ*-secreting cells using an ELISpot assay. The HVT-HA^UL45–46^-vaccinated group exhibited a robust cellular immune response, generating 675 ± 105 spot-forming cells (SFCs) per 10^6^ splenocytes. This response was highly significantly stronger than that induced by the HVT-HA^US2^, which yielded only 243 ± 26 SFCs/10^6^ cells (*p* < 0.001) (Figure 4). Background levels of IFN-*γ* secretion were virtually undetectable in both the MOCK and the unvaccinated PBS groups.

### Protective efficacy against H9N2 AIV challenge

To evaluate the protective efficacy provided by the recombinant vaccines, virus shedding from the oropharyngeal and cloacal swabs was monitored at 3 and 5 dpc. Following Liu et al. [22] and Yang et al. [16], a relatively low-challenge dose of 10^4^ EID_50_ was selected. According to the shedding results after challenge, 100% of the chickens in PBS groups were shedding, which proves that the challenge model is successful at this dose. In the HVT-HA^UL45–46^ group, no virus was detected in cloacal swabs (0/10) at either 3 or 5 dpc. In contrast, cloacal shedding was observed in the HVT-HA^US2^ group, with positive rates of 20% (2/10) and 40% (4/10) at 3 and 5 dpc, respectively. At 3 dpc, the oropharyngeal shedding rates for HVT-HA^UL45–46^, HVT-HA^US2^, and PBS groups were 70% (7/10), 90% (9/10), and 90% (9/10), respectively. At 5 dpc, while the PBS group reached 100% (10/10) oropharyngeal positivity, the HVT-HA^UL45–46^ group showed a reduction in shedding frequency to 60% (6/10), whereas the HVT-HA^US2^ group remained higher at 80% (8/10) (Table 1).

**Table 1:** **Virus shedding detection after challenge with H9N2**

Groups	3 dpc		5 dpc	
	O	C	O	C
HVT-HA ^UL45–46^	7/10	0/10	6/10	0/10
HVT-HA ^US2^	9/10	2/10	8/10	4/10
PBS	9/10	1/10	10/10	1/10

*O* oropharyngeal, *C* cloacal, *dpc* days post-challenge

**Figure 5:**
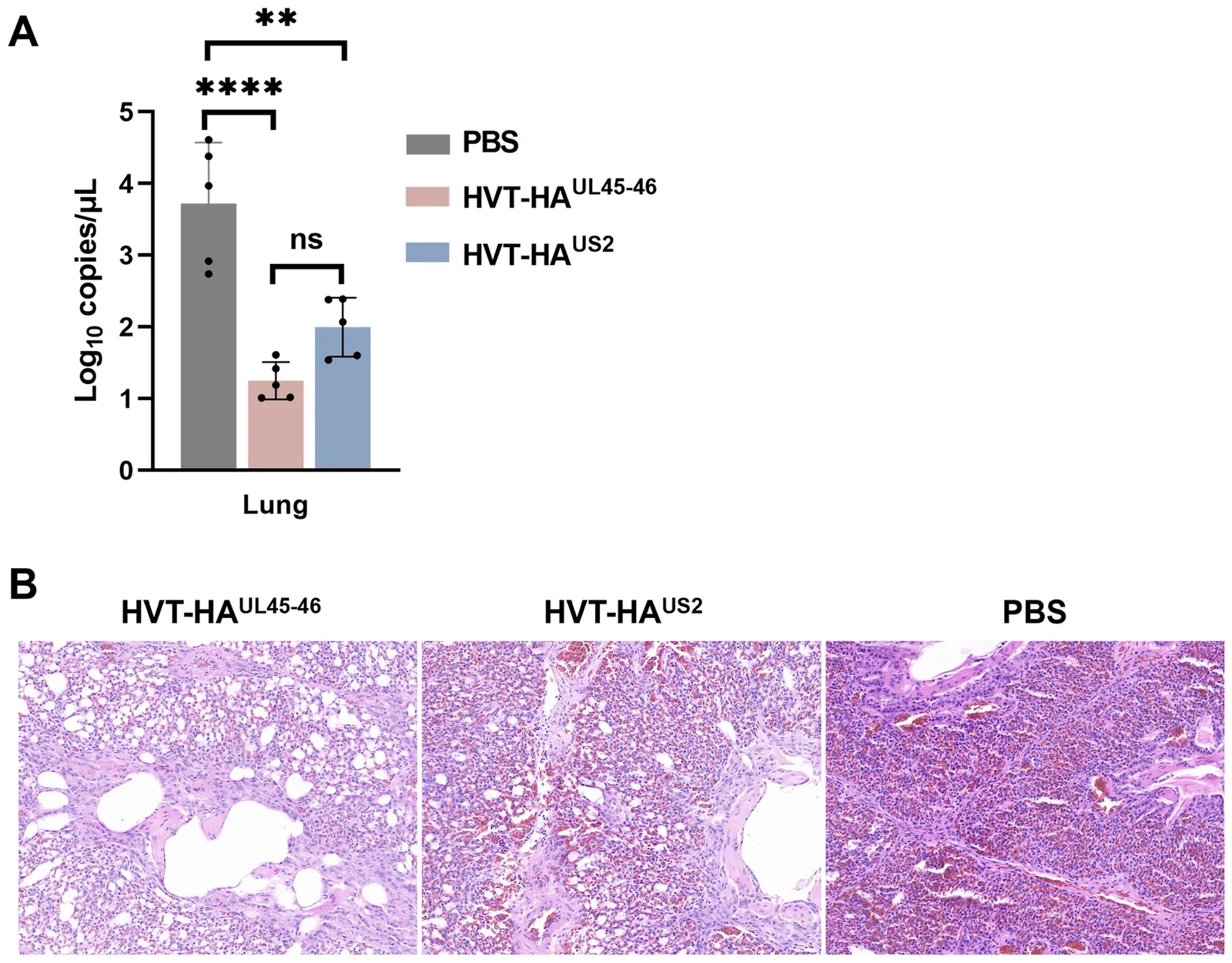
**Lung viral load and histopathological changes in chickens immunized with different HVT-HA vaccines after H9N2 challenge.** **A** Viral loads in lung tissues were determined by quantitative reverse transcription polymerase chain reaction (qRT-PCR) at 5 dpc. Data are presented as mean ± SD (*n* = 5 per group). Statistical significance was analyzed by one-way ANOVA with Tukey’s multiple comparison test (^**^p < 0.01, ^****^p < 0.0001, *ns* not significant). **B** H&E-stained lung tissue sections (× 200 magnification) from each group.

To assess the protective efficacy of the HVT-HA vaccines, lung tissues were collected post-challenge to determine viral loads and examine histopathological changes. Quantitative analysis of the viral titers revealed that the unimmunized control group (PBS) sustained high levels of viral replication in the lungs. In contrast, immunization with both HVT-HA^US2^ and HVT-HA^UL45–46^ significantly reduced lung viral titers compared with the PBS control. Furthermore, a comparison between the two vaccine groups indicated that the HVT-HA^UL45–46^ group exhibited lower viral loads than the HVT-HA^US2^ group, but there was no significant difference (Figure 5A). The PBS group displayed severe lesions, as determined by H&E, characterized by extensive hemorrhage in the alveolar spaces, severe congestion, and inflammatory cell infiltration. Lungs of the HVT-HA^US2^ group still showed some degree of lesions, including marked pulmonary hemorrhage and inflammatory infiltration. The severity was reduced compared with the PBS group. However, the lung lesions were less severe in the HVT-HA^UL45–46^ group. Despite minor inflammatory changes, the lung architecture was well preserved, and hemorrhage and congestion were markedly reduced (Figure 5B).

## Discussion

Recombinant HVT-based vector vaccines have been widely used in poultry because of their strong safety profile, long-term antigen expression, and ability to induce both humoral and cellular immune responses [23, 24]. The development of recombinant HVT expressing the HA gene of AIV has shown to be a potential effective strategy for poultry vaccination [25]. The choice of insertion site is critical because it affects the transcriptional efficiency of the foreign gene and the replication kinetics of the HVT vector [20]. The US2 site is a classical site used in generations of HVT vaccines. However, research has indicated that modifications of some herpes viruses in the US region may affect the in vivo adaptability of the virus or its ability to stimulate T-cell activation [26, 27]. Recent molecular evidence indicates the envelope protein UL46 of alpha herpesviruses HSV-1 is a potent immune antagonist that downregulates TANK-binding kinase 1 (TBK1)-dependent antiviral innate immunity. UL46 has been shown to interact with TBK1 and inhibit its dimerization, thereby impairing the TBK1–IRF3 signaling axis and inhibiting type I interferons’ (IFN-I) production. HVT belongs to the Alphaherpesvirinae family and may have similar characteristics with HSV-1. The disruption or modulation of the UL45–46 region during HA insertion may alleviate this innate immune evasive mechanism, potentially enhancing the adjuvant effect of the vector and promoting more efficient antigen presentation [28]. Therefore, UL45–46 was selected as the insertion site of HA and compared with the US site in this study.

In this study, we demonstrated that both HVT-HA^UL45–46^ and HVT-HA^US2^ elicited robust systemic and mucosal antibody responses. The HI antibody titers gradually increased over time in both vaccinated groups. The HVT vaccine could establish a lifelong persistent infection in vivo, providing sustained endogenous antigen presentation to the host immune system [24, 29]. At 28 dpi, the HVT-HA^UL45–46^ group induced significantly higher HI titers than the HVT-HA^US2^ group. It is possible that the UL45–46 site provides a more favorable transcriptional microenvironment for HA expression than the US2 site, thereby enhancing specific immunogenicity. Interestingly, while HI antibody levels continued to rise, serum NAb titers peaked at 7 dpi and subsequently declined. We will continue to explore the reasons for this phenomenon. Furthermore, our study confirmed the successful induction of mucosal NAbs in the harderian glands, trachea, and duodenum. Conventional inactivated whole-virus vaccines mainly elicited systemic IgG but poorly local mucosal IgA stimulation. Live HVT-vectored vaccines can efficiently activate the mucosal immune system. The presence of local NAbs in the respiratory and intestinal tract is essential for clearance of the H9N2 virus at the primary sites of infection [16].

Cell-mediated immunity (CMI) is essential for the rapid clearance of intracellular viral infections and broad cross-protection against AIV variants [30, 31]. In this study, we employed the IFN-*γ* ELISpot assay to quantitatively assess CMI elicited by two recombinant HVT-H9 vaccines. The results showed that the HVT-HA^UL45–46^ group produced 675 ± 105 SFCs/10^6^ splenocytes, which was significantly higher than that of the HVT-HA^US2^ group (243 ± 26 SFCs/10^6^). These data suggest that the UL45–46 site is the preferred site for the cellular immune potential of HVT-vectored vaccines.

Evaluation of viral shedding following H9N2 challenge revealed that the HVT-HA vaccine using the UL45–46 site provided stronger inhibitory effect on virus replication than the US2 site. HVT-HA^UL45–46^ completely inhibited cloacal shedding at both 3 and 5 dpc. In contrast, the HVT-HA^US2^ group failed to effectively prevent intestinal infection, with cloacal shedding rates reaching 20% and 40% at 3 and 5 dpc, respectively. Although oropharyngeal shedding persisted in all vaccinated groups, reflecting the predominant respiratory tropism of H9N2, the frequency of shedding in the HVT-HA^UL45–46^ group gradually decreased from 70% (3 dpc) to 60% (5 dpc). It was significantly better than the HVT-HA^US2^ (from 90% to 80%) and the PBS control group (from 90% to 100%). These data indicate that the insertion site is a key factor influencing HVT-HA immune efficacy and inhibiting shedding. Chickens immunized with HVT-HA^UL45–46^ elicited significantly higher HI antibody titers and HA-specific cellular response compared with HVT-HA^US2^. Elevated circulating HI antibodies may contribute to neutralize the progeny virus, thereby limiting transmission from blood to the intestinal mucosa. Concurrently, the enhanced cellular response promotes the rapid clearance of infected cells [32, 33].

This study demonstrates that the UL45–46 insertion site in the HVT vector exhibits superior immunogenicity and protective efficacy against H9N2 AIV compared with the US2 site. The HVT-HA^UL45–46^ vaccine maintained robust viral replication kinetics and genetic stability through 20 consecutive passages. It also induced significantly higher HI titers (3.58 ± 0.54 log_2_ at 28 dpi) and HA-specific cellular immunity (675 ± 105 IFN-*γ*-secreting cells/10^6^ splenocytes) than HVT-HA^US2^. Critically, immunization with the HVT-HA^UL45–46^ group completely prevented intestinal viral shedding (0/10 at 3 and 5 dpc) and significantly reduced lung viral loads and histopathological lesions post-challenge. These results identified UL45–46 as the optimal insertion site for HVT-based H9N2 vaccines. This strategy provides a promising platform for next-generation poultry vaccines targeting endemic H9N2 strains.

## Supplementary information

**Additional file 1** **PCR primers for recombinant virus identification**.

**Additional file 2** **PCR identification results of HVT-HA recombinant virus after 20 passages.** CMV enhancer: Cytomegalovirus enhancer. CMV promoter: Cytomegalovirus promoter. EGFP: Enhanced Green Fluorescent Protein. SV40 poly (A) signal: Simian Vacuolating Virus 40 polyadenylation signal. Mcmv iel: Murine Cytomegalovirus Immediate Early 1, IE1. HA: Hemagglutinin.

## Data Availability

No datasets were generated or analyzed during the current study.
