# Supplementary material for: UL45–46 as the preferred insertion site for herpesvirus-of-turkeys-vectored H9N2 avian influenza vaccines: comparative analysis of immunogenicity and protection
Source: Vet Res. 2026 Nov 13;57:201. doi: 10.1186/s13567-026-01850-7 (PMC13760770; doi:10.1186/s13567-026-01850-7)
Supplement: Supplementary file 1 [file 13567_2026_1850_MOESM1_ESM.docx]

Table S1 PCR primers for recombinant virus identification

| Primers | Sequence |
| --- | --- |
| UL45-46-F | TCGAGTCCTCCACCAGCTTA |
| UL45-46-R | ATAAAATTACTGCCAAAGCGAA |
