# Supplementary material for: UL45–46 as the preferred insertion site for herpesvirus-of-turkeys-vectored H9N2 avian influenza vaccines: comparative analysis of immunogenicity and protection
Source: Vet Res. 2026 Nov 13;57:201. doi: 10.1186/s13567-026-01850-7 (PMC13760770; doi:10.1186/s13567-026-01850-7)
Supplement: Supplementary file 2 [file 13567_2026_1850_MOESM2_ESM.docx]

**Figure S1 PCR identification results of HVT-HA recombinant virus after 20 passages.** CMV enhancer: Cytomegalovirus enhancer. CMV promoter: Cytomegalovirus promoter. EGFP: Enhanced Green Fluorescent Protein. SV40 poly (A) signal: Simian Vacuolating Virus 40 polyadenylation signal. Mcmv iel: Murine Cytomegalovirus Immediate Early 1, IE1. HA: Hemagglutinin.
